# Peripheral 5-HT_1A_ and 5-HT_7_ Serotonergic Receptors Modulate Parasympathetic Neurotransmission in Long-Term Diabetic Rats

**DOI:** 10.1155/2010/686734

**Published:** 2011-02-17

**Authors:** Beatriz Restrepo, María Luisa Martín, Luis San Román, Asunción Morán

**Affiliations:** ^1^Laboratorio de Farmacología, Departamento de Fisiología y Farmacología, Facultad de Farmacia, Universidad de Salamanca, Campus Miguel de Unamuno, 37007 Salamanca, Spain; ^2^Grupo de Enfermedades Cardiovasculares y Metabólicas, Facultad de Ciencias de la Salud, Universidad del Quindío, Carrera 15, Calle 12N, Armenia, Quindío, Colombia

## Abstract

We analyzed the modulation of serotonin on the bradycardia induced *in vivo* by vagal electrical stimulation in alloxan-induced long-term diabetic rats. Bolus intravenous administration of serotonin had a dual effect on the bradycardia induced either by vagal stimulation or exogenous Ach, increasing it at low doses and decreasing it at high doses of 5-hydroxytryptamine (5-HT), effect reproduced by 5-carboxamidotryptamine maleate (5-CT), a 5-HT_1/7_ agonist. The enhancement of the bradycardia at low doses of 5-CT was reproduced by 5-HT_1A_ agonist 8-hydroxy-2-dipropylaminotetralin hydrobromide (8-OH-DPAT) and abolished by WAY-100,635, 5-HT_1A_ antagonist. Pretreatment with 5-HT_1_ antagonist methiothepin blocked the stimulatory and inhibitory effect of 5-CT, whereas pimozide, 5-HT_7_ antagonist, only abolished 5-CT inhibitory action. In conclusion, long-term diabetes elicits changes in the subtype of the 5-HT receptor involved in modulation of vagally induced bradycardia. Activation of the 5-HT_1A_ receptors induces enhancement, whereas attenuation is due to 5-HT_7_ receptor activation. This 5-HT dual effect occurs at pre- and postjunctional levels.

## 1. Introduction

The role of 5-hydroxytryptamine (5-HT) in cholinergic neurotransmission and parasympathetic cardiovascular effects has received considerable attention over many years. Central 5-HT_1A_, 5-HT_3_, and 5-HT_7_ receptors have a physiological role in the regulation of cardiovascular reflexes, controlling changes in parasympathetic (vagal) drive to the heart [1]. Recently, Ni et al. [2] revealed the existence of a local 5-hydroxytryptaminergic system in peripheral arteries, but the physiological role of 5-HT in the regulation of vascular tone remains unclear. Some studies have proposed potent depolarizing actions, with an increase in vagal activity mediated by activation of 5-HT_3_ and 5-HT_2_ [3], 5-HT_4_ [4], or 5-HT_1A_ receptors in different animal species [5–9]. Other reports have suggested an inhibitory serotonergic effect on acetylcholine (Ach) release via activation of 5-HT_1_ receptors [10].

We have shown that, in pithed rats, the serotonergic mechanisms involved in cardiac cholinergic neurotransmission are presynaptic and that they can dually inhibit or facilitate acetylcholine release via activation of 5-HT_2_ or 5HT_3_ receptors, respectively [11]. We have also shown that experimental diabetes elicits changes in the nature and 5-HT receptor type/subtype involved in vagal bradycardia stimulated by electrical means [12]. 

Diabetes mellitus (DM) is considered to be an important public health problem owing to its increasing prevalence and because it causes and exacerbates macro- and microvascular complications. These are associated with severe debilitating complications that include a diabetic autonomic neuropathy characterized in part by impaired cardiac parasympathetic responsiveness. Such parasympathetic dysfunction in the diabetic heart may lead to an autonomic imbalance that may predispose the heart to ventricular arrhythmias and sudden death [13]. Long-term diabetes reduces the body's ability to finely regulate heart rate (HR); this is attributed to profound disturbances in autonomic function [14] which are probably due to parasympathetic dysfunction [12, 15]. Several studies conducted under different conditions have supported the theory that, during the development of diabetes, there are impaired myocardial responses to muscarinic activation by acetylcholine. These responses are related to hyper- or hyposensitization and may include changes in expression of muscarinic receptors [16–18]. Other studies have suggested that diabetes induces changes in cholinergic transmission [15, 19]. 

The aim of the present study was to analyze the possible changes induced by experimental long-term diabetes to 5-HT on the *in vivo* cardiac response to activation of parasympathetic nerves. This was achieved by examining the serotonergic receptors involved in the cholinergic cardiac responses to vagal electrical stimulation or by the administration of exogenous acetylcholine during experimental alloxan-induced diabetes.

## 2. Material and Methods

The housing conditions of the rats and experimental procedures were in accordance with regulations set by the European Union on the use of animal for scientific purposes (86/609/EEC, Article 5, Appendix II) and enacted by Spanish legislation on March 14, 1988 (R.D.223/1988).

### 2.1. Animal Preparation

Two hundred fifty-five male Wistar rats (250–350 g) were used in the present study. Rats were supplied and maintained by the Animalarium of the Faculty of Pharmacy at the University of Salamanca (Spain; PAE-SA001). Diabetes was induced by a single injection of alloxan (150 mg/kg, s.c.) dissolved in 0.9% NaCl. Rats were maintained on tap water, and food was available *ad libitum* for 8 weeks. A second group of animals were kept as control normoglycaemic group for eight weeks. Body weight, systolic blood pressure, heart rate, and blood glucose levels were determined before and at 2, 7, 14, 21, 28, 35, 42, 49, and 56 days after alloxan administration. Only rats with elevated blood glucose levels (>11 mM) at all time points were considered to be diabetic. Blood glucose levels were determined by test strips (Accu-Chek). Systolic blood pressure and heart rate were measured in awake rats periodically using the tail-cuff method with a photoelectric sensor (NIPREM 546, Cibertec S.A., Madrid, Spain). Several determinations were made in each session for each rat. Values were considered to be valid if five consecutive measurements were within 10 mmHg.

Eight-week diabetic rats and four-week diabetic rats were anaesthetized with sodium pentobarbital (60 mg/kg, i.p.). After cannulation of the trachea, rats were pithed by inserting a stainless steel rod through the orbit and foramen magnum [20]. They were then artificially respirated with room air using a Harvard respiratory pump (1 mL air/100 g, 50 strokes/min; Harvard Apparatus, South Natick, MA, USA). The right and left jugular veins were cannulated for the infusion of agonists and antagonists, respectively.

Arterial blood pressure was monitored from the left carotid artery cannula using a pressure transducer connected to a PRS 206 amplifier (Cibertec). Heart rate was measured by analyses of blood pressure data with a CAR 306 cardiotachograph (Cibertec). Data on blood pressure and heart rate were shown on a Letica Polygraph 4000 (Cibertec).

Both vagus nerves were isolated, ligated at the cervical level, and cut rostrally to the ligature to prevent afferent and efferent vagal reflexes. Electrical stimulation was applied as square wave pulses from a Cibertec Stimulator CS-9 (supramaximal intensity: 15 ± 3 V; 1 msec; 3, 6, and 9 Hz for 15 s at 5-min intervals) with a platinum bipolar electrode connected to the caudal stump of the right cervical vagus nerve.

Before electrical stimulation, rats were treated with heparin (1000 UI/kg). Then, they were given D-tubocurarine (2 mg/kg, i.v.), to avoid electrically induced muscular twitching, and atenolol (1 mg/kg, i.v.), to prevent sympathetic effects. Rats were kept warm (37.5 ± 0.5°C) with a heating lamp.

### 2.2. Experimental Protocols

After the hemodynamic status of the rats had been stable for ≥10 min, baseline values for mean blood pressure (MBP) and heart rate were determined.

A platinum bipolar electrode was applied to the caudal stump of the right cervical vagus nerve and electrical stimulation applied as square wave pulses from a Cibertec stimulator applying trains of 15 s which consisted of pulses of 1 msec duration and supramaximal intensity (15 ± 3 V) at increasing frequencies (3, 6, and 9 Hz). Thus, the control stimulation response curve was completed in ~15 min. At this point, rats were divided into seventh agonist or antagonist treatment groups (*n* = 5 rats/group).

The first group of experiments was conducted in normoglycaemic (kept in parallel during eight weeks) and four-week diabetic rats to confirm previous data from our research team [11, 12]. Each group was subdivided in two. The first subgroup received an i.v. bolus of saline solution (1 mL/kg; *n* = 5) as a control for that groups of experiments or 5-HT (5, 10, 50, 100, and 200 *μ*g/kg, *n* = 5 for each dose). Five minutes after the treatment, a new stimulation response curve was obtained. In the second subgroup (*n* = 10), the effects of saline solution (1 mL/kg; *n* = 5) or 5-HT (100 *μ*g/kg, *n* = 5) on the bradycardia induced by exogenous acetylcholine (1, 5, and 10 *μ*g/kg) were investigated. In this group, acetylcholine was administered before (control curve) and 5 min after pretreatment with saline solution or 5-HT. 

In the first long-term alloxan-treated diabetic group (*n* = 80), rats received an i.v. bolus of one of the following: (i) saline solution (1 mL/kg, *n* = 5, control group for all the agonist treatments); (ii) 5-HT at 5, 10, 50, 100, and 200 *μ*g/kg (*n* = 25); (iii) 5-carboxamidotryptamine maleate (5-CT; selective 5-HT_1/7_ receptor agonist) at 5, 10, 50, 100, and 200 *μ*g/kg (*n* = 25); (iv) 150 *μ*g/kg of *α*-methyl-5-hydroxytryptamine (selective 5-HT_2_ receptor agonist *n* = 5); (v) 150 *μ*g/kg of 1-phenylbiguanide (selective 5-HT_3_ receptor agonist; *n* = 5); (vi) 50 *μ*g/kg of 8-hydroxydipropylaminotretalin hydrobromide (8-OH-DPAT; selective 5-HT_1A_ receptor agonist; *n* = 5); (vii) 50 *μ*g/kg of CGS-12066B (agonist of the rodent 5-HT_1B_ receptor; *n* = 5); (viii) 50 *μ*g/kg of L-694,247 (selective agonist for nonrodent 5-HT_1B_ and 5-HT_1D_ receptors; *n* = 5). Five minutes after the corresponding administration, a new stimulation response curve was obtained.

The second long-term alloxan-treated diabetic group (*n* = 30) was run in parallel with the group described above. The effect of saline solution (1 mL/kg), 100 *μ*g/kg of methiothepin (*n* = 5), a nonselective 5-HT_1_ receptor antagonist, or 100 *μ*g/kg of WAY-100,635 (*n* = 5), a selective 5-HT_1A_ receptor antagonist, or 1 mg/kg of GR55562 (*n* = 5), a selective 5-HT_1B_ receptor antagonist, or 1 mg/kg of BRL-15572 (*n* = 5), a selective 5-HT_1D_ receptor antagonist, and 1 mg/kg of pimozide (*n* = 5), a selective 5-HT_7_ receptor antagonist, was observed.

The third long-term alloxan-treated diabetic group (*n* = 25) was used to determine which receptor subtype was involved in regulating the effect of 5-HT on heart rate. Methiothepin (100 *μ*g/kg), WAY-100,635 (100 *μ*g/kg), GR55562 (1 mg/kg), BRL-15572 (1 mg/kg), or pimozide (1 mg/kg) were, respectively, administered 5 min before 5-CT (10, 100 *μ*g/kg, *n* = 10), 8-OH-DPAT (50 *μ*g/kg, *n* = 5), CGS-12066B (50 *μ*g/kg, *n* = 5), or L-694,247 (50 *μ*g/kg, *n* = 5), respectively. Five minutes after each treatment, a new stimulation response curve was obtained.

In the fourth group, rats received an i.v. bolus of atropine (0.3 or 0.5 mg/kg, *n* = 5 for each dose) before electrical stimulation to confirm that cholinergic responses were induced by electrical stimulation.

In the final group of long-term diabetic rats (*n* = 30), the effects of saline solution (1 mL/kg; *n* = 5), 5-HT (10 and 100 *μ*g/kg, *n* = 5 for each dose), 5-CT (10 and 100 *μ*g/kg, *n* = 5 for each dose), and 8-OH-DPAT (50 *μ*g/kg, *n* = 5) were examined on the bradycardia induced by exogenous acetylcholine (1, 5, and 10 *μ*g/kg), which was administered before (control curve) and 5 min after drug pretreatment.

### 2.3. Drugs Used

The anaesthetic (pentobarbital sodium) was obtained from Sigma-Aldrich, (St. Louis, MO, USA). Heparin sodium was from Roche (Madrid, Spain). Alloxan and d-tubocurarine hydrochloride were purchased from Sigma-Aldrich. 5-hydroxytryptamine creatinine sulphate complex (5-HT), methiothepin mesylate, 5-carboxamidotryptamine maleate (5-CT), 7-trifluoromethyl-4-(4-methyl-1-piperazinyl)pyrrolo[1,2-a-]-quinoxaline dimaleate (CGS-12066B dimaleate), 2-[5-[3-(4-methylsulfonylamino)benzyl-1,2,4-oxadiazol-5-yl]-1H-indol-3-yl]ethanamine (L-694,247), *α*-methyl-5-hydroxytryptamine maleate (*α*-methyl-5-HT), 1-[1-[4,4-Bis(4-fluorophenyl)butyl]-4-piperidinyl]-1,3-dihydro-2H-benzimidazol-2-one (pimozide), (S)-N-ter-butyl-3-(4-(2-methoxyphenyl)-piperozin-1-yl)-2-phenylpropanamide dihydrochloride (WAY-100,635), 8-hydroxy-2-dipropylaminotetralin hydrobromide (8-OH-DPAT), and 3-[4-(4-chlorophenyl) piperazin-1-yl]-1,1-diphenyl-2-propanol hydrochloride (BRL-12572) hydrochloride were purchased from Tocris Cookson Limited (Ellisville, MO, USA). Atropine sulphate was from Scharlau (Barcelona, Spain), and atenolol was from Sigma-Aldrich. 

All drugs used were dissolved in distilled water at the time of experimentation, with the exception of BRL-15572 (dissolved in 20% propylene glycol) and pimozide (dissolved in 0.01 M HCl).

### 2.4. Statistical Analyses

Data are mean ± SEM of at least five experiments (*n* = 5). Comparison of results between experimental groups and their corresponding control group were undertaken using ANOVA followed by the Newman-Keuls multiple comparison test. Differences were considered to be statistically significant if *P* < .05.

## 3. Results

### 3.1. Systemic Hemodynamic Variables

Alloxan-induced diabetes elicited a marked increase in serum glucose levels and systolic blood pressure. Diabetic rats failed to increase their body weight compared with control rats.  Table 1 shows the mean values of body weight, systolic blood pressure, heart rate, and glycaemia before and 8 weeks after the induction of diabetes for rats in the diabetic group and in the control group.

Resting blood pressure and heart rate were 43.2 ± 2 mmHg and 270 ± 4.9 beats per minute, respectively, in eight-week diabetic anaesthetized pithed rats and 40.5 ± 1.5 mmHg and 290 ± 5.0 beats per minute in anaesthetized four-week diabetic rats. These values were not altered significantly by the i.v. administration of a saline bolus, 5-HT receptor agonists (5-HT, 5-CT, 8-OH-DPAT, CGS-12066B, L-694,247, *α*-methyl-5-HT, and 1-phenylbiguanide) or the 5-HT receptor antagonists (methiothepin, pimozide, BRL-15572, GR55562, and WAY-100,635) (data not shown). 

### 3.2. Effects of Physiological Saline or 5-HT on Vagally Induced Bradycardia in Normoglycaemic and Four-Week Diabetic Rats

Electrical stimulation of the right vagus nerve in normoglycaemic and four-week diabetic rats resulted in frequency-dependent bradycardia. In both groups the decrease in heart rate remained stable after i.v. administration of a bolus of saline solution (1 mL/kg).

**Figure 1:**
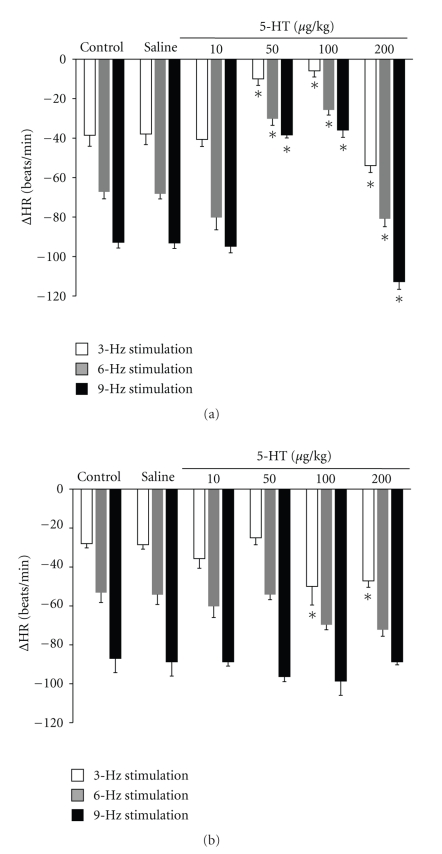
Change in heart rate response (ΔHR) evoked by electrical stimulation of the peripheral end of the right vagus in atenolol (1 mg/kg), pretreated (a) normoglycaemic and (b) four-week diabetic pithed rats before (control; *n* = 5) and after i.v. administration of a bolus of 1 mL/kg saline solution or 10, 50, 100, 200 *μ*g/kg of 5-hydroxytryptamine (5-HT) to normoglycaemic rats (*n* = 5) or 10, 50, 100, 200 *μ*g/kg of 5-HT to four-week diabetic rats (*n* = 5). *P < .05 compared with saline (control).

In normoglycaemic rats, the lowest doses of 5-HT (5, 10 *μ*g/kg, *n* = 10) did not modify the vagally induced bradycardia at the frequencies tested; the administration of 50 and 100 *μ*g/kg (*n* = 10) caused an attenuation of the bradycardia, and the highest dose of 5-HT (200 *μ*g/kg, *n* = 5) resulted in an increase of the vagally induced bradycardia (Figure 1(a)). 

In four-week diabetic rats, the lowest doses of 5-HT (10 and 50 *μ*g/kg, *n* = 10) did not significantly modify the vagally induced bradycardia in the stimulation frequency range tested (Figure 1(b)). However, high doses of 5-HT (100 and 200 *μ*g/kg, *n* = 10) caused an increase in the bradycardia, although this was only significant at the stimulation frequency of 3 Hz (Figure 1(b)).

The stimulatory actions on vagal stimulation-induced bradycardia in diabetic rats were also observed after administration of exogenous acetylcholine (data not shown).

### 3.3. Effects of Physiological Saline or 5-HT on Vagally Induced Bradycardia in Long-Term Diabetic Rats

**Figure 2:**
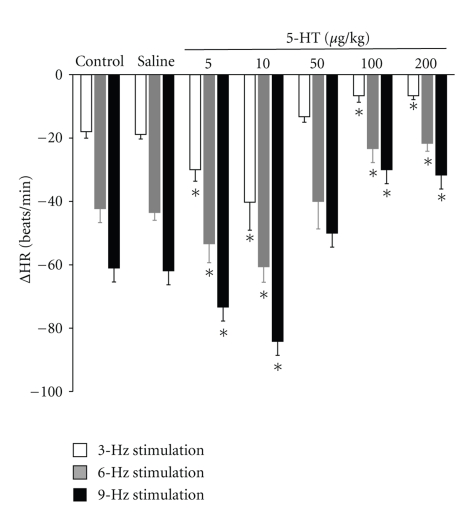
Changes in heart rate (ΔHR) evoked by electrical stimulation of the peripheral end of the right vagus in atenolol (1 mg/kg) pretreated long-term diabetic pithed rats after i.v. administration of a bolus of 1 mL/kg of saline solution, 5, 10, 50, 100, and 200 *μ*g/kg of 5-HT. *P < .05 compared with saline.

 Electrical stimulation of the right vagus nerve in diabetic rats resulted in frequency-dependent bradycardia. This electrically-induced bradycardia, in the long-term diabetic rats, was less pronounced than the bradycardia obtained under identical conditions in four-week diabetic rats. These differences were statistically significant at all stimulation frequencies (3, 6, and 9 Hz) (Figure 2). The decrease in heart rate remained stable after i.v. bolus administration of saline solution (1 mL/kg, *n* = 5). However, these effects caused by electrical stimulation of the vagus nerve were completely blocked by prior administration of atropine (0.5 mg/kg, *n* = 10) (data not shown), confirming the cholinergic nature of the responses to electrical stimulation.

Low doses of 5-HT (5 and 10 *μ*g/kg, *n* = 10) caused a significant increase in the vagally induced bradycardia at all stimulation frequencies tested (Figure 2). However, high doses of 5-HT (100 and 200 *μ*g/kg, *n* = 10) caused a significant decrease in bradycardia at all stimulation frequencies tested (Figure 2).

### 3.4. Effects of i.v. Bolus Administration of 5-HT Receptor Agonists (5-CT, 8-OH-DPAT, CGS-12066B, L-694,247, *α*-methyl-5-HT, and 1-phenylbiguanide) on Vagally Induced Bradycardia in Long-Term Diabetic Rats

**Figure 3:**
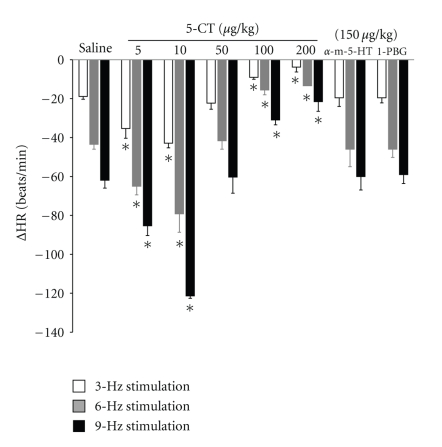
Changes in heart rate (ΔHR) evoked by electrical stimulation of the peripheral end of the right vagus in atenolol (1 mg/kg) pretreated diabetic pithed rats after i.v. administration of a bolus of 1 mL/kg of saline solution, 5, 50, and 200 *μ*g/kg of 5-carboxamidotryptamine (5-CT; *n* = 5 for each dose), *α*-methyl-5-HT (*α*-m-5-HT; 150 *μ*g/kg), or 1-phenylbiguanide (1-PBG; 150 *μ*g/kg). *P < .05 compared with saline.

In eight-week diabetic rats, the bradycardic effects induced by vagal electrical stimulation were modified by 5-CT (a selective 5-HT_1/7_ receptor agonist) depending on the dose administered. At low doses (5 and 10 *μ*g/kg), 5-CT enhanced electrically induced bradycardia (Figure 3), whereas high doses (50, 100, and 200 *μ*g/kg) inhibited electrically induced bradycardia (Figure 3).

**Figure 4:**
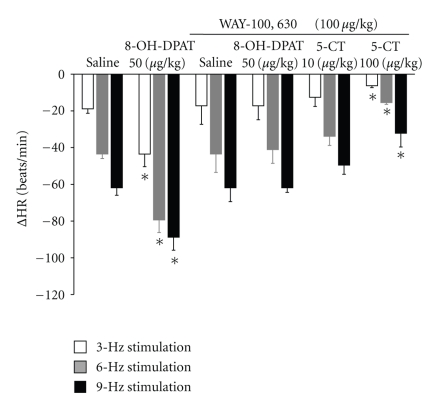
Changes in heart rate (ΔHR) after i.v. administration of a bolus of 8-hydroxydipropylaminotretalin hydrobromide (8-OH-DPAT, 50 *μ*g/kg; *n* = 5) and response after i.v. administration of a bolus of WAY-100,635 (100 *μ*g/kg) on the effect of 8-hydroxydipropylaminotretalin hydrobromide (8-OH-DPAT, 50 *μ*g/kg; *n* = 5) and 5-carboxamidotryptamine (5-CT, 10, 100 *μ*g/kg; *n* = 10) on bradycardia induced by vagal electrical stimulation in diabetic pithed rats. *P < .05 compared with saline.

The enhanced bradycardic effect induced by low doses of 5-CT was reproduced by administration of the selective 5-HT_1A_ receptor agonist, 8-OH-DPAT (50 *μ*g/kg) (Figure 4). This effect was significant at all stimulation frequencies tested. However, i.v. bolus administration of the selective 5-HT_1B_ receptor agonist, CGS-12066B (50 *μ*g/kg), and the selective 5-HT_1D_ receptor agonist, L-694,247 (50 *μ*g/kg), had no effect on the bradycardic responses evoked by electrical stimulation of the vagus nerve (data not shown). Administration of the selective 5-HT_2_ receptor agonist, *α*-methyl-5-HT (150 *μ*g/kg), or selective 5-HT_3_ receptor agonist, 1-phenylbiguanide (150 *μ*g/kg), did not modify the decreases in heart rate induced by vagal electrical stimulation (Figure 3).

### 3.5. Effect of i.v. Bolus Administration of 5-HT Receptor Antagonists (Methiothepin, WAY-100,635, and Pimozide) on Vagally Induced Bradycardia in Long-Term Diabetic Rats

In eight-week diabetic pithed rats, administration of either the selective 5-HT_1A_ receptor antagonist, WAY-100,635 (100 *μ*g/kg, *n* = 5), or the nonselective 5-HT_1_ receptor antagonist methiothepin (100 *μ*g/kg, *n* = 5) or the selective 5-HT_7_ receptor antagonist, pimozide (1 mg/kg, *n* = 5), did not significantly modify the heart rate induced by vagal electrical stimulation (data not shown). 

**Figure 5:**
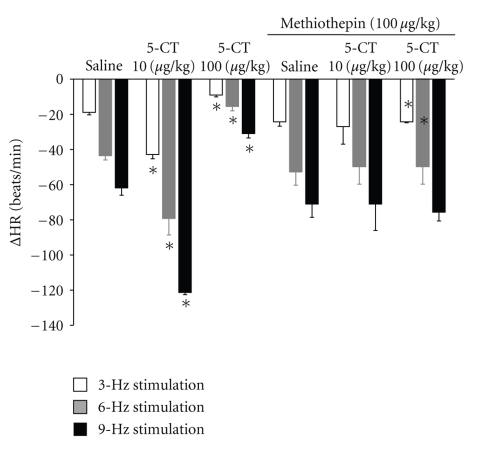
Changes in heart rate (ΔHR) after i.v. administration of a bolus of 5-carboxamidotryptamine (5-CT, 10, 100 *μ*g/kg) and response after i.v. administration of a bolus of methiothepin (100 *μ*g/kg) on the enhanced and inhibitory effect of 5-CT (10, 100 *μ*g/kg) on bradycardia by vagal electrical stimulation in diabetic pithed rats. *P < .05 compared with saline.

Pretreatment with methiothepin (100 *μ*g/kg) blocked the potentiating and inhibitory effect of 5-CT on the bradycardia induced by vagal stimulation in diabetic rats (Figure 5).

Pretreatment with WAY-100,635 (100 *μ*g/kg) reduced the enhancement of the bradycardic effect caused by low doses of 5-CT (10 *μ*g/kg; Figure 3) and abolished the effects of 8-OH-DPAT (50 *μ*g/kg, *n* = 5; Figure 4).

**Figure 6:**
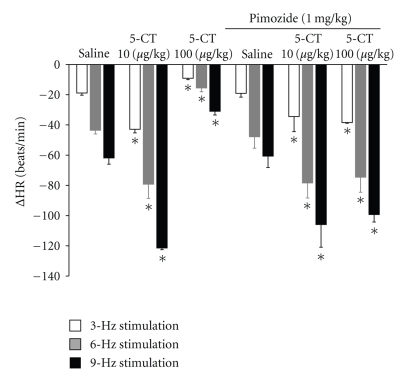
Changes in heart rate (ΔHR) after i.v. administration of a bolus of 5-carboxamidotryptamine (5-CT, 10, 100 *μ*g/kg) and response after i.v. administration of a bolus of pimozide (1 mg/kg) on the enhanced effect and inhibitory effect of 5-CT (10, 100 *μ*g/kg) on bradycardia by vagal electrical stimulation in diabetic pithed rats. *P < .05 compared with saline.

Administration of the selective 5-HT_7_ receptor antagonist pimozide (1 mg/kg) did not modify the potentiating effect of the lowest 5-CT doses (10 *μ*g/kg, *n* = 5; Figure 6). However, it abolished the inhibitory action of high doses of 5-CT (100 *μ*g/kg, *n* = 5; Figure 6).

### 3.6. Effects of Saline, 5-HT, 5-CT, and 8-OH-DPAT on the Bradycardia Induced by Exogenous Acetylcholine Administration in Long-Term Diabetic Rats

**Figure 7:**
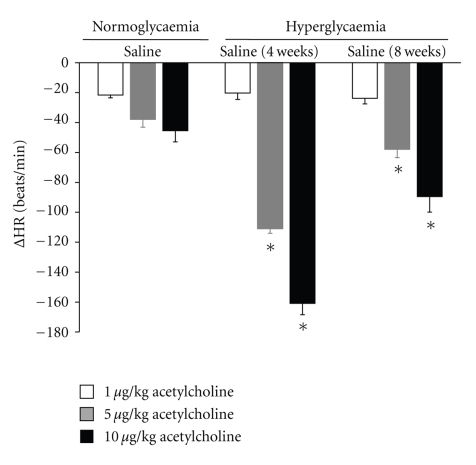
Changes in heart rate (ΔHR) evoked by exogenous administration of 1, 5, and 10 *μ*g/kg acetylcholine in atenolol (1 mg/kg) pretreated in normoglycaemic, four-week, and long-term diabetic pithed rats after i.v. administration of a bolus of 1 mL/kg. *P < .05 compared with normoglycaemic group.

 Exogenous administration of acetylcholine (5 and 10 *μ*g/kg) in long-term hyperglycemic rats produced a significantly increased bradycardia compared with normoglycemic rats. However, the bradycardia was less pronounced than in short-term diabetic rats (Figure 7).

**Figure 8:**
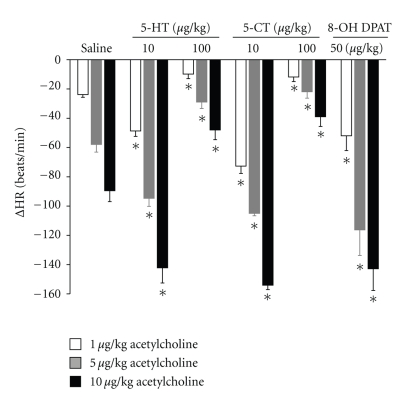
Changes in heart rate (ΔHR) evoked by exogenous administration of 1, 5, and 10 *μ*g/kg acetylcholine in atenolol (1 mg/kg) pretreated in long-term diabetic pithed rats after i.v. administration of a bolus of 1 mL/kg of saline solution, 5-hydroxytryptamine (5-HT, 10 and 100 *μ*g/kg; *n* = 10), 5-carboxamidotryptamine (5-CT 10 and 100 *μ*g/kg; *n* = 10), or 8-hydroxydipropylaminotretalin hydrobromide (8-OH-DPAT 50 *μ*g/kg; *n* = 5). *P < .05 compared with saline.

In a group of rats, bradycardia was induced by exogenous administration of the muscarinic agonist acetylcholine (1, 5, and 10 *μ*g/kg), resulting in dose-dependent decreases in heart rate. These bradycardic effects, in eight-week diabetic rats, remained stable after i.v. bolus administration of saline (1 mL/kg, *n* = 5). However, administration of 5-HT (10 *μ*g/kg, *n* = 5), 5-CT (10 *μ*g/kg, *n* = 5), or 8-OH-DPAT (50 *μ*g/kg, *n* = 5) enhanced the bradycardia, whereas i.v. bolus administration of higher doses of 5-HT (100 *μ*g/kg, *n* = 5) or 5-CT (100 *μ*g/kg, *n* = 5) inhibited the bradycardia induced by administration of exogenous acetylcholine (Figure 8). 

## 4. Discussion

In the present study, we examined the changes induced by experimental long-term diabetes to 5-HT on the *in vivo* cardiac response to activation of parasympathetic nerves. This was achieved by examining the serotonergic receptors involved in the cholinergic cardiac responses induced by vagal electrical stimulation or by the administration of exogenous acetylcholine during experimental alloxan-induced diabetes. 

Alloxan is a diabetogenic agent which induces a syndrome in animals resembling type 1 diabetes mellitus characterized by hyperglycemia, hypercholesterolemia, glycosuria, and raised levels of glycosylated hemoglobin in erythrocytes [12, 17, 21–24]. This agent, in our experiments, did not modify heart rate, as previously indicated by Lee et al. [25], Zola et al. [26], and Howarth et al. [27].

Herein, we showed that there was no significant difference in basal heart rate among the three study groups (normoglycemic, long-term, and short-term diabetic rats) as reported by us and others [11, 12, 28]. This is in contrast with reports that describe a reduced intrinsic heart rate [29] and parasympathetic tonus in diabetes [30].

We showed that, in long-term diabetic rats, the bradycardic effect produced by electrical stimulation of the vagus nerve (at all stimulation frequencies) was lower than the observed in short-term diabetic rats and was impaired compared with normoglycemic rats [11, 12]. In 2007, Ago et al. [31], using diabetic rats, showed an increase in heart rate induced by vagal stimulation using stimulation frequencies higher than those used by us (16, 32, and 64 Hz).

We also demonstrated that administration of exogenous acetylcholine (5 and 10 *μ*g/kg) in long-term hyperglycaemic rats produced a more pronounced bradycardia than in normoglycaemic rats (statistically significant difference). However, the bradycardia was less pronounced than in short-term diabetic rats. Our research team and others have suggested that chemically induced diabetes may elicit functional defects in cardiac cholinergic nerves [11, 32–34]. In the heart, inhibitory M_2_ muscarinic receptors on the nerves limit acetylcholine release [35–37]. The increased inhibition of acetylcholine release by inhibitory neuronal M_2_ muscarinic receptors during experimental diabetes has been described extensively in different experimental models, including rat lungs [16], the ileum and trachea from diabetic rats [18], the urinary bladder [38], and even in human cardiac atrium [39]. Therefore, in the present study, we examined the changes induced by long-term diabetes in the action that 5-HT exerts on the *in vivo* cardiac response to parasympathetic nerves activation. 

5-HT is the well-characterized endogenous ligand for all 5-HT receptors. 5-HT had a dual effect on the bradycardia induced by electrical stimulation in experimental long-term diabetic rats. This dual effect was reproduced by the selective 5-HT_1/7_ receptor agonist 5-CT [40]: at low doses (5 and 10 *μ*g/kg), 5-HT and 5-CT increased the bradycardia, whereas higher doses (100 and 200 *μ*g/kg) of 5-HT or 5-CT decreased the bradycardic effect at all stimulation frequencies. In contrast, administration of *α*-methyl-5-HT (a selective 5-HT_2A/2B/2C_ receptor agonist) [41] or 1-phenylbiguanide (a selective 5-HT_3_ receptor agonist) [42, 43] had no effect on vagally induced bradycardia in diabetic rats. These findings suggest that, in experimental long-term (eight-week) diabetes, the serotonergic effects on bradycardia are mediated through activation of the 5-HT_1_ receptors but not through activation of the 5-HT_2_ or 5-HT_3_ receptors. We had previously demonstrated that, in normoglycemic pithed rats, 5-HT_2_ receptors are involved in the inhibition of vagally induced bradycardia and that the 5-HT_3_ receptor is required for stimulatory action [11]. Therefore, we propose that, in experimental diabetes, as in normoglycaemia [11, 12], the serotonergic system interferes with cholinergic cardiac transmission, producing enhancement and inhibition of the bradycardic effect induced by vagal stimulation.

Several authors have hypothesized that, at gastrointestinal level, in normoglycaemic animals, 5-HT_1_ receptors are involved in the reduction of acetylcholine release [10, 44]. To determine which 5-HT_1_ receptor subtype is responsible for the action observed in this paper, we used antagonists and selective 5-HT_1_ agonists in our experimental model of diabetes.

In long-term diabetic rats, we noted that the increases observed in the vagally induced bradycardia after administration of low doses of 5-HT and 5-CT (selective 5-HT_1/7_ receptor agonist) were mimicked, at all stimulation frequencies, by the selective 5-HT_1A_ receptor agonist 8-OH-DPAT [45]. 

However, and unlike the effects observed in four-week diabetic rats, L-694,247, a selective agonist for nonrodent 5-HT_1B_ and 5-HT_1D_ receptors [46], failed to mimic the inhibitory action on vagally induced bradycardia elicited by high doses of 5-CT. Also, CGS-12066B, a rodent 5-HT_1B_ receptor agonist [47], had no effect on vagally induced bradycardia. These findings suggest that the 5-HT_1A_ receptor subtype is involved in regulating the stimulatory effect observed. 

Regarding the antagonists utilized, the selective 5-HT_1A_ antagonist WAY-100,635 [48] and the selective 5-HT_7_ receptor antagonist pimozide [49] did not affect the vagally induced bradycardia in long-term diabetic rats. However, the vagally induced bradycardia was slightly enhanced by methiothepin (nonselective 5-HT_1_ receptor antagonist) [50]. These findings are consistent with reports describing an intrinsic effect on acetylcholine release in rat striatal slices for methiothepin [51].

We have previously indicated a dual effect for 5-CT in four-week diabetic rats [12]. This effect was reproduced in eight-week diabetic rats, but the receptor subtype involved in the inhibitory effect was different. Furthermore, in the present study we showed that, in long-term diabetic rats (8 weeks), (i) methiothepin [40], a nonselective 5-HT receptor antagonist, blocked the enhanced and inhibitory effects of 5-CT on the vagally induced bradycardia; (ii) 8-OH-DPAT (selective 5-HT_1A_ receptor agonist) could enhance the bradycardia produced by vagal stimulation; (iii) the action of 8-OH-DPAT was abrogated by WAY-100,635 (selective 5-HT_1A_ antagonist) [52]; (iv) pimozide (selective 5-HT_7_ antagonist) [49] blocked the inhibitory effect of high doses of 5-CT; (v) after pretreatment with pimozide, 5-CT could increase the bradycardia produced by vagal stimulation at all doses tested.

 Diabetes mellitus is associated with severe debilitating complications that include diabetic autonomic neuropathy characterized in part by impaired cardiac parasympathetic responsiveness [13]. Parasympathetic dysfunction in the diabetic heart may lead to an autonomic imbalance which may predispose the heart to ventricular arrhythmias and sudden death. Our findings suggest a possible peripheral action of serotonergic receptors in modulating cholinergic transmission, in addition to the well-known central 5-HT_1A_ receptor regulation in cardiovascular effects [7, 53]. Also, the role of 5-HT_7_ receptors in facilitating vagal outflow activation to the heart [1, 8, 54] has been suggested. 

We also showed that the dual effect (stimulatory and inhibitory actions) of the vagally induced bradycardia caused by 5-HT and 5-CT persisted if the bradycardia was elicited by exogenous acetylcholine in long-term diabetic rats. 

These results confirm the pre- and postjunctional nature of these serotonergic actions in long-term diabetes. Nevertheless, these findings are in contrast with results described by our research team in normoglycaemic rats [11] and were similar to those found in four-week diabetic pithed rats [12].

The present study showed that long-term diabetes changed the responses to 5-HT on the *in vivo* cardiac response to activation of parasympathetic nerves. Peripheral 5-HT_1A_ and 5-HT_7_ receptors may have a physiological role in the regulation of cardiovascular reflexes, controlling changes in parasympathetic (vagal) drive to the heart. In this line, in the nucleus tractus solitaries, Oskutyte et al. [55] have reported the presence of 5-HT_1A_ and 5-HT_7_ receptors, which play an important role in cardiovascular reflex activation of parasympathetic outflow to the heart.

In conclusion, experimental long-term diabetes induces changes in the nature and the type/subtype of 5-HT receptors involved in vagal bradycardia induced by electrical stimulation. Activation of the 5-HT_1A_ receptor subtype induces enhancement of vagally induced bradycardia, whereas attenuation of this bradycardia is due to activation of the 5-HT_7_ receptor subtype. The effects induced by 5-HT occur at the pre- and postjunctional level in long-term diabetic pithed rats. Based on these and our previous results, the alloxan-induced diabetes in rats (short- and long-term diabetes) is an appropriate model to study the role of 5-HT and its receptors in the development and progression of the autonomic and endothelial dysfunction due to diabetes mellitus type 1. Further investigation is warranted to determine whether 5-HT_7_ receptors play a pathophysiological role in the autonomic dysfunction due to this chronic disease.

## Figures and Tables

**Table 1:** Values of body weight, systolic blood pressure, heart rate, and glycaemia in control and diabetic rats.

	Body weight (g)	Systolic blood pressure (mmHg)	Heart rate (bpm)	Glycaemia (mM)	*n*
*Control rats*					
Initial time	219 ± 9.0	118 ± 5.0	375 ± 10.0	5.6 ± 0.2	40
8 weeks after	424 ± 16.0	130 ± 4.0	392 ± 15.0	4.8 ± 0.1	40

*Diabetic rats*					
Initial time	192 ± 10.0	124 ± 5.0	310 ± 6.0	5.6 ± 0.1	175
8 weeks after	309 ± 11.8*	157 ± 0.7*	365 ± 10.0	20.1 ± 0.7*	175

Results are means ± SEM for “*n*” rats.

*Significantly different from the corresponding value in control rats, *P* < .05.
